# Serine mutation of a conserved threonine in the hERG K^+^ channel S6-pore region leads to loss-of-function through trafficking impairment

**DOI:** 10.1016/j.bbrc.2020.04.003

**Published:** 2020-06-11

**Authors:** Ehab Al-Moubarak, Yihong Zhang, Christopher E. Dempsey, Henggui Zhang, Stephen C. Harmer, Jules C. Hancox

**Affiliations:** a School of Physiology, Pharmacology and Neuroscience, Biomedical Sciences Building, University Walk, Bristol, BS8 1TD, UK; b School of Biochemistry, Biomedical Sciences Building, University Walk, Bristol, BS8 1TD, UK; c Biological Physics Group, School of Physics and Astronomy, The University of Manchester, Manchester, M13 9PL, UK

**Keywords:** Arrhythmia, hERG, I_Kr_, Long QT syndrome, LQTS, Rapid delayed rectifier, Trafficking

## Abstract

The *human Ether-à-go-go Related Gene* (*hERG*) encodes a potassium channel responsible for the cardiac rapid delayed rectifier K^+^ current, I_Kr_, which regulates ventricular repolarization. Loss-of-function *hERG* mutations underpin the LQT2 form of congenital long QT syndrome. This study was undertaken to elucidate the functional consequences of a variant of uncertain significance, T634S, located at a highly conserved position at the top of the S6 helix of the hERG channel. Whole-cell patch-clamp recordings were made at 37 °C of hERG current (I_hERG_) from HEK 293 cells expressing wild-type (WT) hERG, WT+T634S and hERG-T634S alone. When the T634S mutation was expressed alone little or no I_hERG_ could be recorded. Co-expressing WT and hERG-T634S suppressed I_hERG_ tails by ∼57% compared to WT alone, without significant alteration of voltage dependent activation of I_hERG_. A similar suppression of I_hERG_ was observed under action potential voltage clamp. Comparable reduction of I_Kr_ in a ventricular AP model delayed repolarization and led to action potential prolongation. A *LI-COR®* based On/In-Cell Western assay showed that cell surface expression of hERG channels in HEK 293 cells was markedly reduced by the T634S mutation, whilst total cellular hERG expression was unaffected, demonstrating impaired trafficking of the hERG-T634S mutant. Incubation with E−4031, but not lumacaftor, rescued defective hERG-T634S channel trafficking and I_hERG_ density. In conclusion, these data identify hERG-T634S as a rescuable trafficking defective mutation that reduces I_Kr_ sufficiently to delay repolarization and, thereby, potentially produce a LQT2 phenotype.

## Highlights

• hERG potassium channel variants can cause dangerous ventricular arrhythmias.
• An S6 helix threonine in hERG, T634, is highly conserved amongst potassium channels.
• The T634S mutation reduces hERG current and its contribution to ventricular repolarization.
• The T634S mutation decreases hERG channel surface expression but not synthesis.
• T634S-induced hERG trafficking impairment is pharmacologically rescuable.

## 1 Introduction

Electrical repolarization determines the duration of ventricular action potentials and, thereby, the length of the QT interval on the electrocardiogram. Of the potassium channel currents that contribute to ventricular repolarization, the rapid delayed rectifier current, I_Kr_, appears to be particularly notable. The *human Ether-à-go-go Related Gene* (*hERG*; alternative nomenclature *KCNH2*) encodes channels that mediate I_Kr_ [1,2]. Loss-of-function *hERG* mutations lead to the LQT2 form of congenital long QT syndrome [3,4], whilst gain-of-function *hERG* mutations underpin the SQT1 form of short QT syndrome [5]. Additionally, the marked susceptibility of hERG channels to pharmacological blockade strongly implicates the channel in cases of acquired (drug-induced) LQTS [3]. Most *hERG* mutations linked to congenital LQT2 are missense mutations, the majority of which impair channel transport within the cell (trafficking); misfolded hERG proteins become retained within the endoplasmic reticulum, thereby limiting the number of functional channels in the cell membrane [4,6]. Over 1000 *hERG* variants exist on publicly available databases such as ClinVar, but functional data are available for only a fraction of these.

**Fig. 1:**
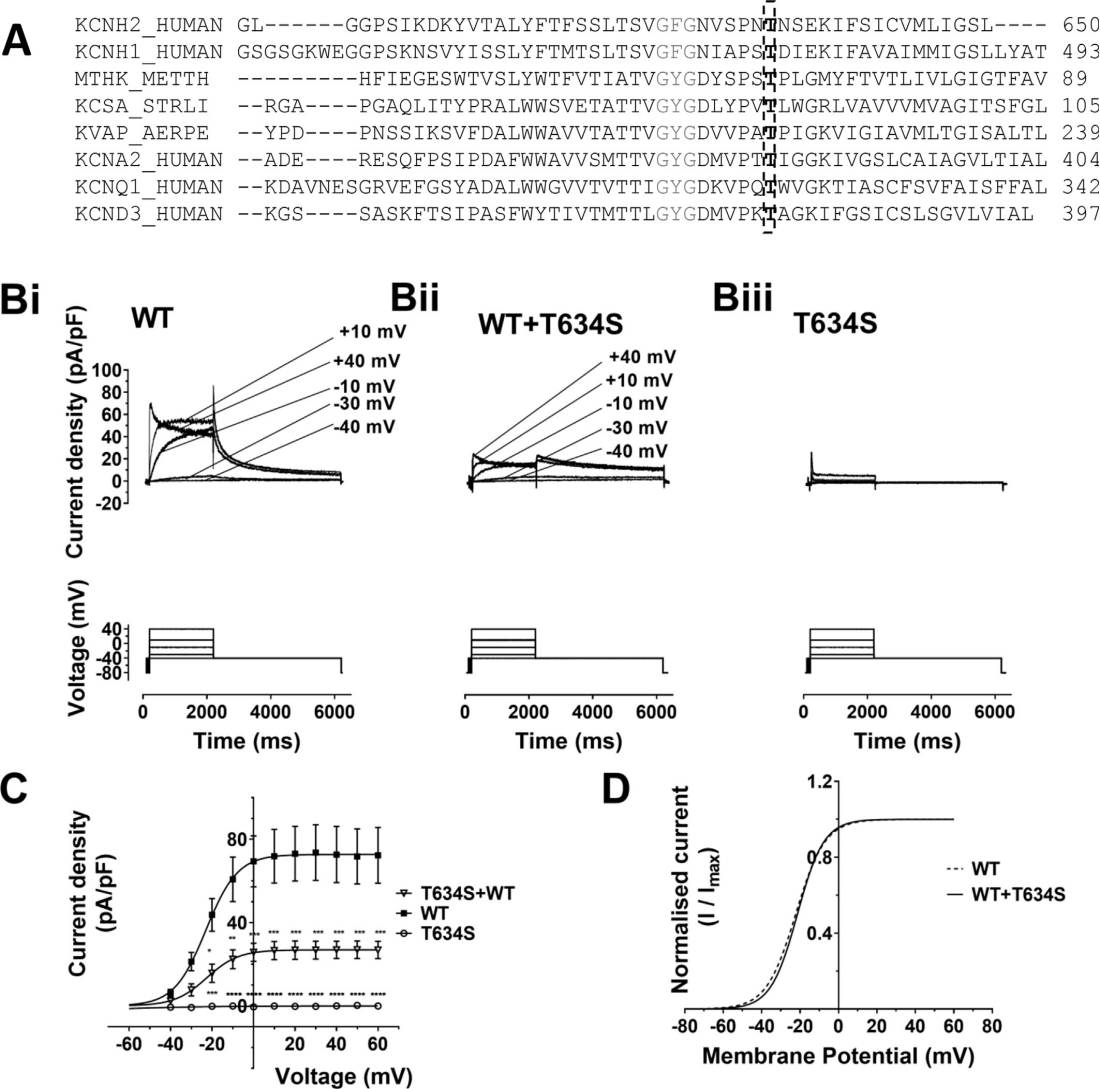
**Sequence alignments and** c**urrent-voltage (I–V) relations for WT, T643S and WT** + **T634S hERG**. **A)** Sequence alignments between hERG (KCNH2), EAG (KCNH1), bacterial (MthK, KcsA, KvAP) and other human (KCNA2-encoded Kv2.1, KCNQ1, and KCND3-encoded Kv4.3) potassium channels, showing conservation across all these channels of the threonine (T) residue at position 634 in hERG. **B)** Representative current (I_hERG_) traces for **Bi**: WT, **Bii**: WT+T634S and **Biii**: T634S hERG. Voltage protocol was comprised of 2s depolarising commands applied at 10 mV increments between −40 mV and +60 mV, from −80 mV (start-to-start interval of 12 s). Only selected traces are shown for clarity. The corresponding voltage commands are indicated to the right of currents in **Bi** and **Bii**. **C):** Mean I–V relations for tail current of WT, T634S, and the WT+T634S I_hERG_ following each command pulse. The plots for WT and WT+T634 hERG were fitted with a modified Boltzmann equation. **D)**: Derived activation curve for WT (dashed line) and WT and T634S (continuous line). V_0.5_ for WT: 22.3 ± 1.0 mV, *k*: 7.6 ± 0.5 (n = 7). V_0.5_ for WT+T634S: 21.4 ± 2.9 mV, *k*: 6.9 ± 0.3 (n = 5). Asterisks denote significant difference from corresponding value for WT I_hERG_ (∗ = P < 0.05; ∗∗P < 0.01; ∗∗∗P < 0.001; ∗∗∗∗P < 0.0001).

LQT2 associated mutations in the transmembrane pore region of hERG appear to be associated with a higher risk of arrhythmia events than those in other regions of the channel [7,8]. Pathogenicity cannot automatically be assumed, however, as some variants may be benign. Patch clamp used together with a biochemical assay of hERG channel expression has been demonstrated to have significant value for classifying *hERG* variants of uncertain significance (VUS) [9,10]. A threonine residue (T634) at the top of the S6 helix of the hERG channel has recently been identified as able to hydrogen-bond with a glutamate (E575) at the top of the S5 segment and to comprise part of a hydrogen-bonded network of residues that forms a ring around the top of the channel’s selectivity filter [11]. This threonine residue is highly conserved amongst potassium channels (Fig. 1A). An LQT2 associated isoleucine mutation at this position (T634I) has previously been reported to lead to defective hERG channel trafficking [6] and a second mutation (T634A) has been reported in an adolescent LQTS patient diagnosed by a school-based screening program [12]. The present study was undertaken to characterize a novel hERG VUS, T634S, providing the first functional data on any mutation at this position in the hERG protein. The results demonstrate that T634S leads to a marked, but pharmacologically rescuable, trafficking defect.

## 2 Materials and methods

### 2.1 Identification and production of the T634S hERG mutation

A c.1901C > G base transition, leading to a missense (p.T634S) mutation was reported anonymized [13] as a VUS by a regional clinical genetics service. Use of the polymorphism phenotyping informatics tool “PolyPhen-2” (http://genetics.bwh.harvard.edu/pph2/) evaluated this mutation as ‘probably damaging’, whilst the “Mutation assessor” tool (http://mutationassessor.org/r3/) predicted it to have medium functional impact. The T634S hERG and T634S HA-tagged hERG mutations were generated using the QuikChange® II site-directed mutagenesis kit (Agilent Technologies) and confirmed using Sanger sequencing. Further details are given in the online supplementary Methods.

### 2.2 Electrophysiological recording

For electrophysiological experiments, HEK 293 cells were transiently transfected with WT and/or hERG-T634S cDNAs, with Lipofectamine following the manufacturer’s instructions, using CD8 as a marker of successful transfection [14]. The total amount of hERG cDNA transfected (1 μg) was kept constant; thus for WT+T634S conditions the amount of each construct transfected was half that used when each channel was expressed alone. Recordings were made at 37 °C using whole cell patch clamp, as described previously [14]. Further information is given in the online supplementary Methods. Mathematical modelling of the consequences of the reduction in I_Kr_ magnitude due to the T634S mutation was performed using the O’Hara-Virag-Varro-Rudy human action potential model [15], reducing gKr by 57.1% to match experimentally observed reduction in I_hERG_ when the WT and T634S hERG channels were co-expressed.

### 2.3 On/In-Cell Western evaluation of hERG expression

*LI-COR*® based Cell Surface (CSA) (‘On-Cell’) and Total cellular hERG expression (Total) (‘In-Cell’) Western assays were combined to evaluate effects of the T634S mutation on hERG channel trafficking in HEK 293 cells. The ‘On-Cell’ Cell Surface Assay (CSA) enabled quantitative monitoring of the level of hERG channel expression at the cell surface. An extracellular epitope was provided by an HA-epitope tag inserted between the S1 and S2 transmembrane domains [16,17] (see red section in Fig. 3Ai). The ‘In-Cell’ Total Assay enabled quantitative monitoring of total cellular hERG channel expression in fixed and permeabilized cells (see Fig. 3Ai). Assays were performed in 48 well assay plates. Each well was transfected, using Lipofectamine 2000, with a total of 1 μg of vector DNA. Where HA-hERG-WT (WT) and HA-hERG-T634S (T634S) were co-transfected, 500 ng of each vector was used (1 μg total). Transfections were performed as detailed in the schematic diagram presented in Fig. 3Aii. Assays were performed 48 h after transfection. Compounds were applied (E−4031 (5 μM), lumacaftor (5 μM) and DMSO) 24 h before assay as indicated in Fig. 3 Aii. Full methodological details are given in the online supplementary Methods.

### 2.4 Drugs

E−4031 was obtained from Tocris (Abingdon, UK); a stock solution of 10 mM was made in distilled, deionized water. Lumacaftor was a gift from Professor David Sheppard (University of Bristol, UK) and was made as a stock solution of 10 mM in DMSO [18]. To evaluate I_hERG_ rescue, these compounds were applied at 5 μM for 24 h before I_hERG_ recording. Cells were washed and kept in drug-free medium for 1–2 h before recording.

### 2.5 Data analysis and statistics

Data are presented as mean ± standard error of the mean (SEM). Statistical analysis was performed using unpaired *t* tests, 1-way or 2-way ANOVA with Bonferroni post-hoc test as appropriate. Details of the statistical tests used to evaluate significance of results for particular experiments are given alongside *P* values either in the main text or relevant figure legend.

## 3 Results and discussion

### 3.1 Effects of the T634S mutation on I_hERG_ during conventional voltage clamp

I_hERG_ recordings were made using a standard voltage protocol comprised of a 2s depolarization from a holding potential of −80 mV to a series of test potentials between −40 mV and +60 mV (in 10 mV increments) followed by repolarization to −40 mV, at which potential I_hERG_ ‘tail’ magnitude was monitored and subsequently normalized to current density (cf [19,20]). Fig. 1Bi-Biii show representative I_hERG_ recordings respectively under WT, WT+T634S and T634S conditions. Co-expression of WT with T634S channels (Fig. 1 Bii) led to a marked reduction in I_hERG_ amplitude across the range of test potentials compared to WT I_hERG_ (Fig. 1Bi). When T634S was expressed alone (Fig. 1 Biii) I_hERG_ ‘tails’ were negligible. Mean normalized tail current data for each expression condition are plotted against corresponding test potential in Fig. 1C. This shows that I_hERG_ tails were suppressed across a wide range of voltages under WT+T634S (heterozygous) conditions compared to the WT channel, whilst they were negligible under T634S (homozygous) expression conditions. The tail I–V relations for WT and WT+T634S I_hERG_ were fitted with a modified Boltzmann equation to yield an activation V_0.5_ for WT I_hERG_ of −22.3 ± 1.0 mV (*k* = 7.6 ± 0.5 mV; n = 7) and for WT+T634S of −21.4 ± 2.9 mV (*k* = 6.9 ± 0.3 mV); n = 5; P > 0.05 for both V_0.5_ and *k*). Fig. 1D shows overlain activation relations for WT and WT+T634S I_hERG_ calculated from the experimentally obtained values. Collectively, the data in Fig. 1B–D shows that T634S led a marked loss of hERG channel function over a wide range of experimental voltages, without a significant shift in voltage-dependent activation of I_hERG_. Supplemental experiments employing a single voltage command (to +20 mV) were used to evaluate effects of the T634S mutation on WT+T634S I_hERG_ deactivation. This followed a biexponential time-course under both conditions; the proportion of deactivating current fitted with fast (τ_f_) and slow (τ_S_) time-constants was similar between the two conditions. However, there was a modest increase in both τ_f_ (from 183.8 ± 26.0 ms; n = 10 to 386.6 ± 95.3 ms; n = 9; P < 0.05) and τ_S_ (from 1333 ± 145 ms; n = 10 to 2522 ± 491 ms; n = 9; P < 0.05). Thus, the dominant effect of the T634S mutation was suppression of I_hERG_ magnitude, with a modest slowing of deactivation time-course.

### 3.2 Evaluation of functional consequences of the T634S mutation

**Fig. 2:**
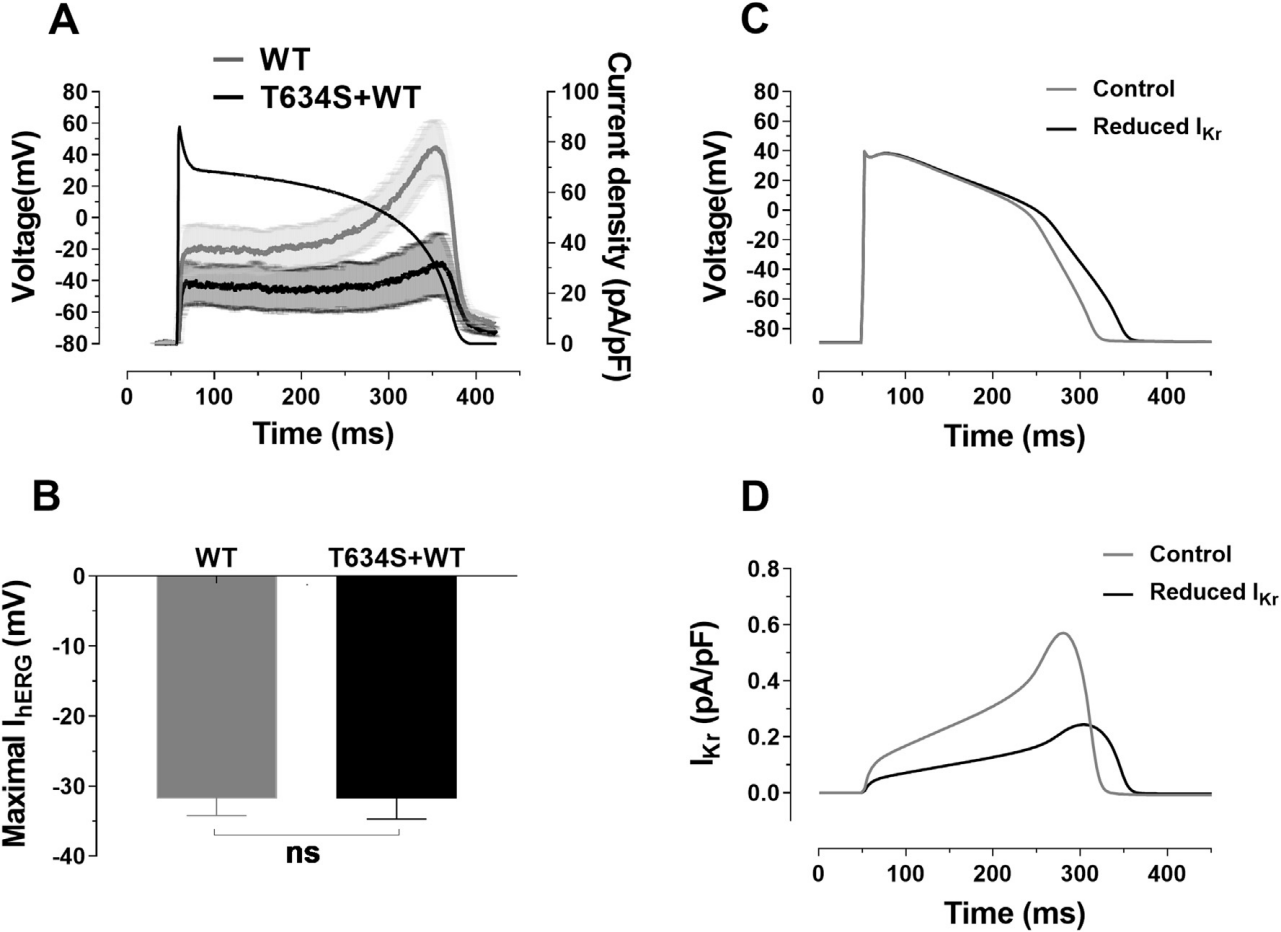
**I**_**hERG**_**during ventricular action potentials: AP clamp and AP simulation**. **A)** Means ± SEM I_hERG_ for WT (n = 5) and WT+T634S conditions (n = 5). Corresponding action potential (AP) command is superimposed on the plotted normalized currents. Co-expression of WT and T634S suppressed current compared to WT hERG alone. **B**) Bar chart comparing the voltage (for WT and WT+T634S) where peak I_hERG_ occurred during AP repolarization, WT (n = 5) and WT+T634S (n = 5; P > 0.05 versus WT, unpaired *t*-test). **C)** Epicardial action potentials from the O’Hara-Virag-Varro-Rudy model generated under control conditions at 1 Hz stimulation frequency and with reduced I_Kr_ (gKr reduced by 57.1%). **D)** Corresponding I_Kr_ records during the control and reduced I_Kr_ APs shown in **C**.

The action potential (AP) voltage-clamp technique enables ionic currents to be elicited by a physiological waveform and therefore exhibit a physiological time-course and voltage-dependence. Fig. 2A shows the mean (±SEM) profile of WT I_hERG_ and WT+T634S I_hERG_ during an applied ventricular AP voltage command, as utilized previously [14]. WT I_hERG_ increased progressively through the AP plateau, peaking just before the rapid terminal phase of repolarization (cf [[19], [20], [21], [22]]). WT+T634S I_hERG_ showed a similar profile to WT I_hERG_, but with current suppressed throughout the AP command. The voltage at which I_hERG_ was maximal during repolarization lay between −30 and −40 mV as previously reported [[19], [20], [21], [22]]) and did not differ between WT and WT+T634S conditions (Fig. 2B).

**Fig. 3:**
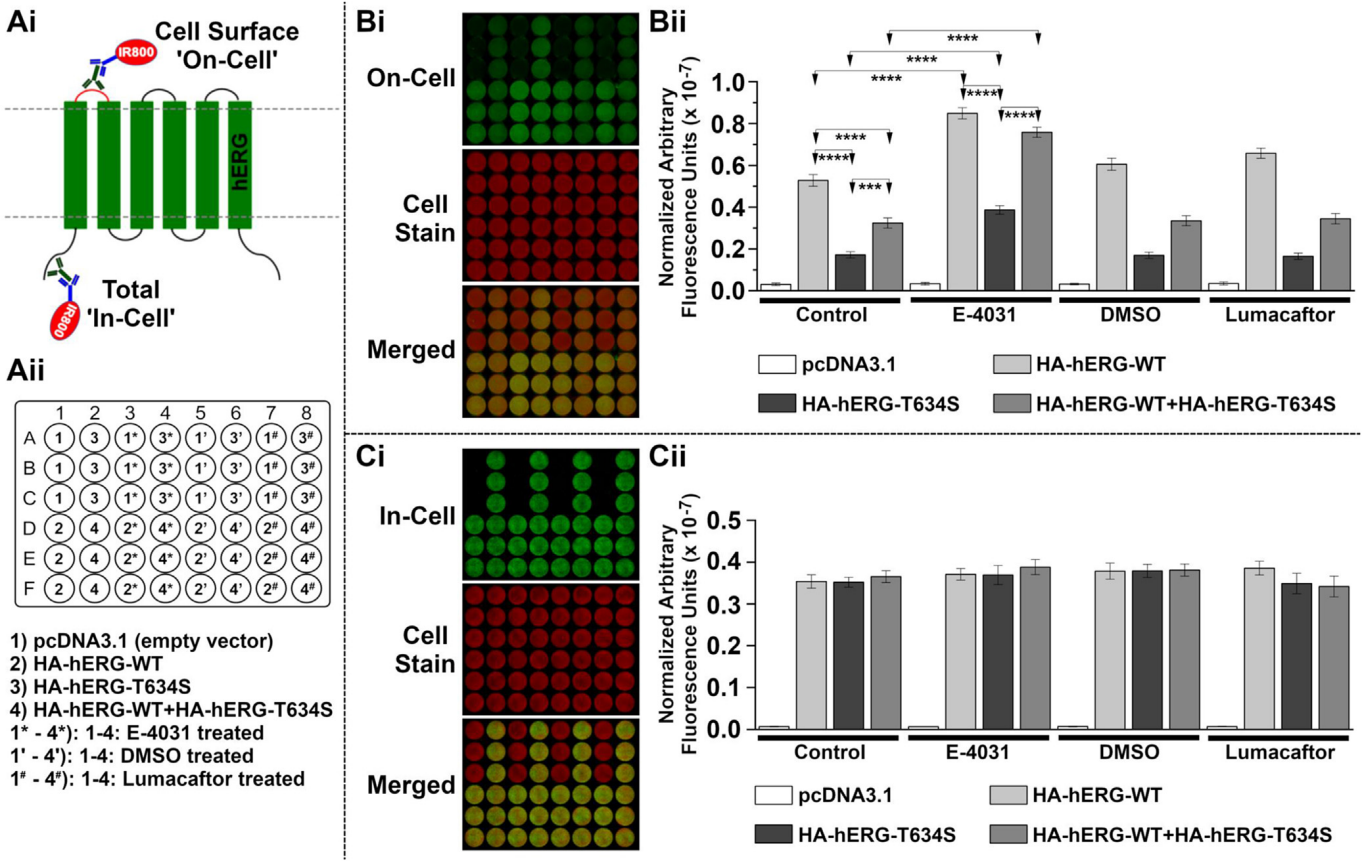
**Effect of T634S mutation on hERG channel trafficking and potential for pharmacological rescue. Ai,ii**) Schematic diagram showing experimental setup for *LI-COR*® based Cell Surface (CSA) (‘On-Cell’) and Total cellular hERG expression (Total) (‘In-Cell’) Western assays. **B**) ‘On-Cell’ Cell Surface hERG expression. Upper panel **Bi** shows a representative Cell Surface (CSA) (‘On-Cell’) Western assay (green channel (800); upper panel). To determine cell number, cells were stained using WGA-680 (red channel (700); middle panel) and the two channels are merged in the lower panel. **Bii**: Quantified Cell Surface ‘On-Cell’ hERG expression levels of WT, T634S, and co-expression of WT+T634S. A 24 h pretreatment with 5 μM E−4031 significantly increased the cell surface expression level of WT, T634S and T634S+WT. In contrast, a 24 h pretreatment with 5 μM lumacaftor did not lead to significant changes in cell surface expression when compared to vehicle control (treatment with DMSO alone). **C**) ‘In-Cell’ Total cellular hERG expression. Upper panel **Ci** shows a representative Total cellular (Total) (‘In-Cell’) Western assay (green channel (800); upper panel). To determine cell number, cells were stained using WGA-680 (red channel (700); middle panel) and the two channels are merged in the lower panel. **Cii**: Quantified Total ‘In-Cell’ cellular hERG expression levels of WT, T634S, and co-expression of WT+T634S in control and 24 h pretreatment with 5 μM E−4031, 5 μM lumacaftor or vehicle control (DMSO treatment was included as a vehicle control for lumacaftor). No significant differences in Total cellular hERG channel expression levels were detected between any of the groups. Statistical analyses were performed using One Way ANOVA and Bonferroni’s multiple comparison; ∗∗∗∗ = P < 0.0001 & ∗∗∗ = P < 0.001. (For interpretation of the references to colour in this figure legend, the reader is referred to the Web version of this article.)

The difference between WT and WT+T634S peak I_hERG_ in Fig. 2A represents a reduction of ∼57% in peak repolarizing current. In order to evaluate consequences of the reduction in functional I_hERG_ on ventricular repolarization, we investigated effects of reduction of I_Kr_ by this proportion in a human ventricular myocyte model [15]. Fig. 2C shows epicardial ventricular APs under control conditions and with decreased I_Kr_. The reduction in I_Kr_ (Fig. 2D), simulating the effect of the T634S mutation (under heterozygotic conditions), led to a lengthening of AP duration at 90% repolarization (APD_90_) from 263 ms to 297 ms (a 34 ms lengthening in APD_90_). Similar simulations were performed for midmyocardial and endocardial cell models (not shown), with respective APD_90_ prolongation observed in midmyocardial and endocardial AP models from 329 to 372 ms (a 43 ms prolongation) and 263–305 ms (a 42 ms prolongation). The difference between epicardial and midmyocardial APD_90_ (a measure of repolarization heterogeneity) in control was 66 ms and with I_Kr_ reduction was 75 ms. Thus, a reduction in I_Kr_ commensurate with the effect of the T634S mutation under heterozygous conditions led both to APD_90_ prolongation and augmented heterogeneity of repolarization between epicardial and midmyocardial cell models. Augmented dispersion of repolarization has been observed in experimental models of LQT2 (e.g. [23,24]) and may produce a substrate favourable to re-entrant arrhythmia.

### 3.3 Impairment of hERG channel trafficking by the T634S mutation

Fig. 3A–C shows the methodology (Fig. 3Ai, Aii see also ‘Methods’) for and representative examples of hERG cell surface and total cell expression as analysed using *LI-COR®* based On/In-Cell Western assays. Each condition shown was repeated in triplicate and the entire assay was repeated on at least 3 separate occasions for all conditions. Visual inspection of Fig. 3Bi shows clear reductions in cell surface expression compared to WT for each of WT+T634S and T634S alone. Fig. 3Bii shows mean (±SEM) normalized data for ‘On-Cell’ cell surface expression. For T634S alone, cell surface expression was greatly reduced (by 67.9%; 0.17 ± 0.02 arbitrary fluorescent units (x 10^−7^)) compared to that of the WT channel (0.53 ± 0.03 units), with co-expression of WT+T634S producing an intermediate level of cell surface expression of 0.32 ± 0.02 units, equating to a 39% reduction (both T634S conditions were significantly lower than those for the WT channel; P < 0.001). Fig. 3Ci shows representative images of ‘In-Cell’ total cell hERG channel expression for the conditions shown in Fig. 3Aii. There was no significant difference in total ‘In-Cell’ hERG channel expression between WT and mutant conditions. Taken collectively, these data indicate that T634S containing channels are synthesized, but not effectively transported to the cell surface membrane. This finding is consistent with an earlier observation that the T634I mutation reduces the amount of mature (fully glycosylated) hERG, indicative of defective trafficking [6].

**Fig. 4:**
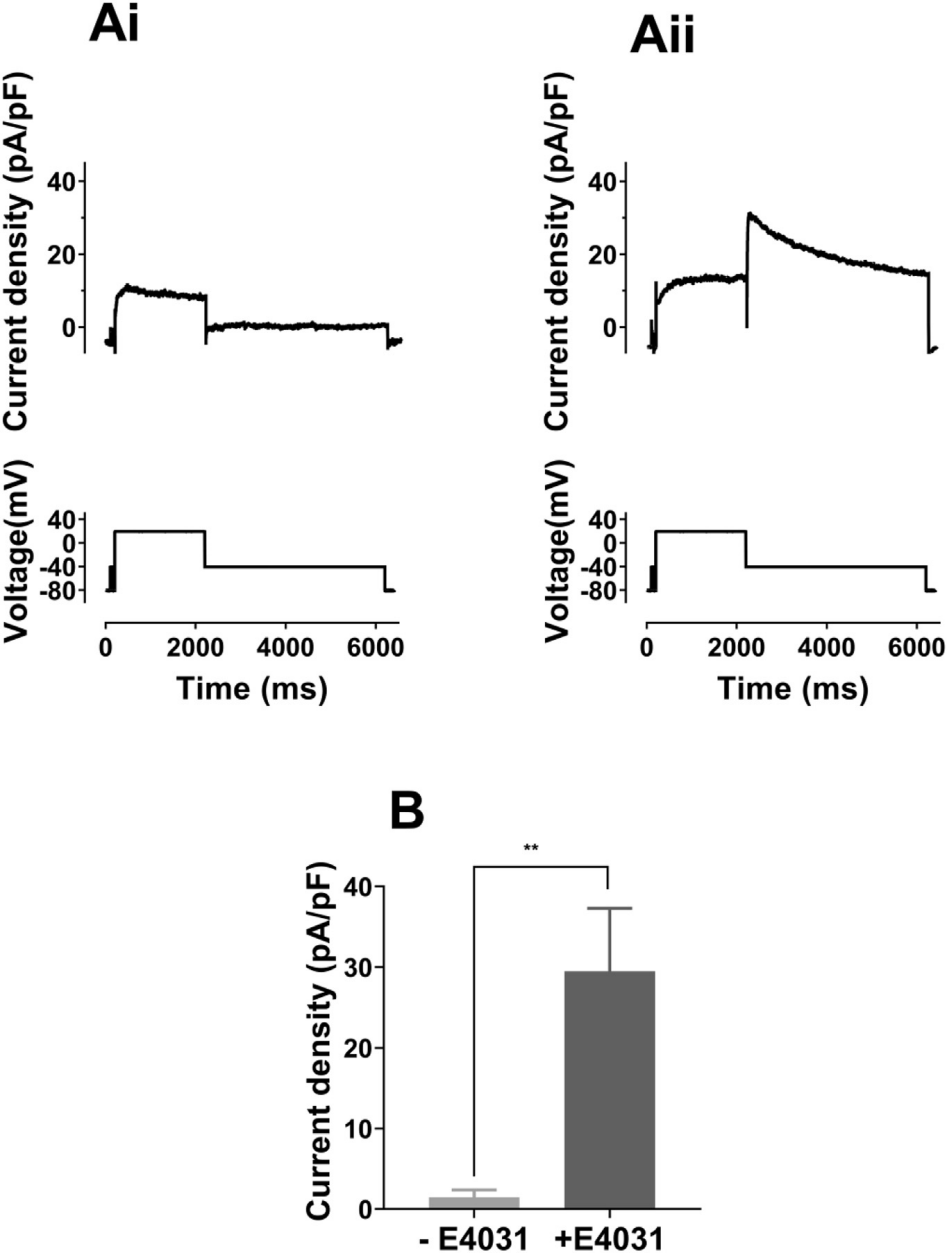
**Functional rescue of T634S I**_**hERG**_**.** (**Ai**,**Aii**) Upper traces are representative examples of current elicited by voltage protocol shown as lower traces for T634S transfected cell without (**Ai**) and following (**Aii**) 24 h incubation in 5 μM E−4031. In each case, recordings were made following a 1–2 h washout of E−4031. (**B**) Mean amplitude of outward current on repolarization to −40 mV for T634S transfected cells that were not exposed to E−4031 (- E−4031; n = 6) and following E−4031 pre-treatment (+E−4031; n = 8).

Some hERG trafficking deficient mutations are amenable to pharmacological rescue ([4,6]). Accordingly, we evaluated whether or not the cell surface expression level of T634S containing channel complexes could be rescued by incubation with 5 μM E−4031. Fig. 3Bi and Ci show representative images of WT, WT+T634S and T634S transfected cells in the absence and presence of E−4031 whilst Fig. 3Bii and 3 Cii incorporates the corresponding mean data. Pretreatment of T634S transfected cells with E−4031 led to a substantial increase in channel surface expression, under conditions mimicking both heterozygotic and homozygotic expression. Surface expression of WT hERG in E−4031 treated cells exceeded that of WT hERG in untreated cells (see Fig. 3Bii), whilst the surface expression level of WT+T634S channels (0.76 ± 0.02 units) was also substantially increased compared to that without E−4031 and that for T634S alone was more than doubled (from: 0.17 ± 0.02 units to 0.39 ± 0.02 units after E−4031; P < 0.0001). We also tested lumacaftor, a CFTR F508del mutation corrector [18] in cystic fibrosis, that has recently been reported to exert beneficial effects on some hERG trafficking defective LQT2 mutants [25]. Surface expression of T634S in 5 μM lumacaftor treated cells did not differ from that in vehicle (DMSO) controls (Fig. 3Bii). Thus, 24 h exposure to E−4031 but not lumacaftor acted to rescue surface expression of T634S. In order to determine whether incubation with E−4031 also led to an increase in expression of *functional* hERG channels, additional experiments were performed in which I_hERG_ was elicited using a depolarising command to +20 mV. Fig. 4Ai and Aii show example traces of recordings from T634S transfected cells without (Fig. 4Ai) and following (Fig. 4 Aii) 24 h of E−4031 preincubation. In the absence of E−4031 treatment, there was an absence of the resurgent tail current that is characteristic of I_hERG_. By contrast, in E−4031 pretreated cells significant I_hERG_ was elicited with a large deactivating I_hERG_ tail. Fig. 4B compares mean I_hERG_ tail density, showing the very large increase in tail current in E−4031 pretreated cells. Taken together with the data in Fig. 3, both surface protein and electrophysiological measurements indicated that functional rescue of T634S channels by E−4031 occurred.

### 3.4 Conclusions – results in context

The positional conservation between different K^+^ channels of a threonine residue at T634 in hERG (Fig. 1A) and also of hydrogen bonding between this threonine and a glutamate residue at the top of the S5 helix [11] indicate that T634 is located in a functionally important region of the hERG channel pore. It is notable that mutations of the analogous residue (T322 → T322A or T322M) in KCNQ1 channels have been linked to the LQT1 form of LQTS, by causing dominant-negative suppression of KCNQ1+KCNE1 (“I_Ks_”) current [26]. Mutations to nearby residues in hERG have been associated with LQTS [27] and the T634I and T634A mutations at the same position in the hERG protein have previously been associated with LQT2 [6,12]. However, the present study is both the first to be conducted on the T634S mutation and the first to contain an electrophysiological characterization of any missense mutation to this residue. It is also the first to directly measure mutation effects at this position on surface expression rather than using mature channel glycosylation as a surrogate marker of surface expression [6]. A combination of similar approaches to those adopted here has recently been shown in a large-scale study to discriminate effectively between benign and pathogenic hERG variants [9]. The effect of the T634S mutation on I_hERG_ deactivation kinetics here was mild and our AP clamp data show that the timing of WT+T634S I_hERG_ during the AP was unaffected by the mutation although current amplitude was markedly reduced. Our findings unambiguously demonstrate that the T634S mutation is detrimental to hERG channel trafficking but not synthesis. This results in a reduction in I_Kr_ under conditions mimicking heterozygous expression that is sufficient to lead to significant ventricular action potential prolongation and, by extension, potentially to an LQT2 phenotype. T634S can therefore be categorized as a ‘Class 2’ (i.e. trafficking) mutation [4]. Under homozygous expression conditions T634I was previously reported to be an uncorrectable trafficking deficient mutation [6]. It is interesting, therefore, that in the present study E−4031 incubation improved T634S trafficking both under WT-mutant co-expression conditions and when the mutation was studied alone. This may suggest that the trafficking dysfunction with T634S is more pharmacologically tractable than is that caused by T634I. However, as the methodologies for evaluating surface expression differ between the earlier study and our own [6], such comparison must be made with caution.

It is important to note that whilst the approaches adopted in this study provide direct insight into mutation-induced channel dysfunction, they cannot provide insight into clinical penetrance of a mutation, nor do they supplant the need for careful characterization of a carrier’s ECG phenotype. However, it is striking that even amongst carriers of LQT2 mutations with normal rate corrected QT (QT_c_) intervals, men carrying hERG pore mutations have a higher risk of cardiac events than those carrying non-pore mutations (hazard ratio 6.01) [28]. This, in turn, highlights the utility of functional characterization of VUS located in the pore region of the hERG channel, so that clear functional and biochemical information can be available for consideration by clinical decision-makers.

## Declaration of competing interest

The authors declare no conflicts of interest.
