# Supplementary material for: Serine mutation of a conserved threonine in the hERG K+ channel S6-pore region leads to loss-of-function through trafficking impairment
Source: Biochem Biophys Res Commun. 2020 Jun 11;526(4):1085–91. doi: 10.1016/j.bbrc.2020.04.003 (PMC7237882; doi:10.1016/j.bbrc.2020.04.003)
Supplement: Multimedia component 1 [file mmc1.pdf]

Serine mutation of a conserved threonine in the hERG K^+ channel S6-pore region leads to loss-of-function through trafficking impairment.

SUPPLEMENTARY ONLINE METHODS

Ehab Al Moubarak[1+], Yihong Zhang[1+], Christopher E Dempsey[2], Henggui Zhang[3], Stephen C Harmer[1*], Jules C Hancox[1*]

[1]School of Physiology, Pharmacology and Neuroscience, Biomedical Sciences Building, University Walk, Bristol, BS8 1TD.

[2]School of Biochemistry, Biomedical Sciences Building, University Walk, Bristol, BS8 1TD.

[3] Biological Physics Group, School of Physics and Astronomy, The University of Manchester, Manchester, M13, 9PL.

[+] these authors contributed equally to this study

[*] authors for correspondence: s.c.harmer@bristol.ac.uk
jules.hancox@bristol.ac.uk

Identification and production of the T634S hERG mutation

A c.1901C>G base transition, leading to a missense (p.T634S) mutation was reported anonymized [1] as a VUS by a regional clinical genetics service. Use of the polymorphism phenotyping informatics tool "PolyPhen-2" (http://genetics.bwh.harvard.edu/pph2/) evaluated this mutation as 'probably damaging', whilst the "Mutation assessor" tool (http://mutationassessor.org/r3/) predicted it to have medium functional impact. The T634S hERG and T634S HA-tagged hERG mutations were generated using the QuikChange® II site-directed mutagenesis kit (Agilent Technologies). In brief, a pair of complementary oligonucleotide primers containing the mutation (forward primer sequence 5'CGTCTCTCCCAACAGCAACTCAGAGAAG3'and reverse primer sequence 5'CTTCTCTGAGTTGCTGTTGGGAGAGACG3'), synthesized by Sigma (Sigma-Aldrich, UK), was used in a PCR reaction (95 °C for 1 min, 60 °C for 1 min, 68 °C for 16 min for 18 cycles) by using hERG in a modified pcDNA3.0 vector and HA-tagged hERG in pcDNA3.1 as the DNA template. The HA-tagged hERG construct [2] was a gift from Professor Alvin Shrier (McGill University, Canada). This construct contains the reference sequence (NM_000238.3, NCBI) with an extracellular HA tag inserted between S1 and S2 domains. A DpnI digest of the PCR mix was then performed for 1 h at 37 °C. Competent DH5*α* *Escherichia coli* (Invitrogen, Paisley, UK) were transformed using standard procedures. The mutations were confirmed by sequencing of the entire open reading frame (Eurofins MWG Operon, Ebersberg, Germany) [3].

Electrophysiological recording

For electrophysiological experiments, HEK 293 cells were transiently transfected with WT and/or hERG-T634S cDNAs, with Lipofectamine following the manufacturer's instructions, using CD8 as a marker of successful transfection [4]. The total amount of hERG cDNA transfected (1 μg) was kept constant; thus for WT+hERG-T634S conditions the amount of each construct transfected was half that used when each channel was expressed alone. Recordings were made at 37°C using whole cell patch clamp, as described previously [3; 4]. Transfected cells were then plated on sterilized coverslip glass shards in 40 mm diameter petri-dishes after a minimum of 6 hours incubation at 37°C (5% CO_2), following transfection. Electrophysiological recordings were made at least 24 hrs after transfection at 37°C, using an Axopatch 200B amplifier (Molecular Devices) with a CV-4/100 headstage and data acquisition via a Digidata 1320 interface (Molecular Devices). Glass shards containing plated cells were

placed in a recording chamber mounted on an inverted microscope (Nikon Diaphot, USA). A standard extracellular Tyrode's superfusate contained (in mM): 140 NaCl, 4 KCl, 2.5 $CaCl_2$, 1 $MgCl_2$, 10 glucose, and 5 HEPES (titrated to pH 7.4 with NaOH) Patch pipettes (A-M Systems Inc, USA) of resistance 2-4 MΩ were filled with a solution containing (in mM): 130 KCl, 1 $MgCl_2$, 5 EGTA, 5 MgATP and 10 HEPES (titrated to pH 7.2 with KOH) [3; 4]. Pipette resistance was typically compensated 60-80%. Recorded currents were filtered at 2 kHz and digitized at 10 kHz.

On/In-Cell Western Assays

For On/In-Cell Western assays HEK 293 cells were cultured on poly-L-lysine coated (P4707, Sigma-Aldrich) 48 well plates (677180, CELLSTAR, Greiner Bio-One). Near confluent cells were transfected using Lipofectamine 2000 (11668, ThermoFisher Scientific) as per manufacturers instructions.

'On-Cell' (Cell Surface expression) Western assay

This assay was used to quantify HA-hERG channel expression at the plasma membrane (PM). 48 hours after transfection, cells were incubated with an anti-HA antibody (mouse monoclonal anti-HA antibody [Sigma Aldrich, H9658]) diluted 1:1000 in ice-cold HEK 293 media at 4 ^oC for 1 hour (250 µl/well). Cells were then washed once in ice-cold HEK 293 media (500 µl/well) followed by two washes in ice- cold PBS^+ (Phosphate Buffered Saline + 1 mM $MgCl_2$ and 0.1 mM $CaCl_2$) (500 µl/well) before being fixed at room temperature in 3.7 % formaldehyde [Sigma Aldrich, 252549] (500 µl/well) for 10 minutes and 10 minutes on a rocking platform. Fixation was followed by 3 washes with ice-cold PBS^+ (500 µl/well). Cells were then washed once with 500 µl/well of HBSS (Hank's Balanced Salt Solution) [Sigma Aldrich, H6648] before adding Wheat Germ Agglutinin 680 Alexa Fluor [Life technologies, W32465] at 5 µg/ml in HBSS (500 µl/well). From this point onwards the cells were protected from light by wrapping the plate in aluminium foil. The plate was then placed on a rocking platform for 10 minutes before being washed twice with PBS^+ (500 µl/well). After washing, the secondary antibody (anti-mouse IgG (H+L) (DyLight 800 conjugate) [New England Biolabs, 5275S]) was diluted 1:1000 in HEK 293 media and 250 µl/well was added. After incubation for 1 hour at room temperature on a rocking platform the cells were washed three times in ice-cold PBS^+ and the assay signal analysed using a *LI-COR*® Odyssey CLx imaging system.

'In-Cell' (Total cellular expression) Western assay

This assay was used to quantify the total amount of HA-hERG channel expression in fixed and permeabilized cells. 48 hours after transfection, cells were washed twice with ice-cold PBS^+ (500 µl/well) and then fixed at room temperature in 3.7 % formaldehyde (500 µl/well) for 10 minutes followed by 10 minutes on a rocking platform. After fixation, cells were washed three times with PBS^+ (500 µl/well) followed by one wash with HBSS (500 µl/well). Cells were then stained with WGA-680 using 5 µg/ml diluted in HBSS (500 µl/well). From this point onwards the cells were protected from light by wrapping the plate in aluminium foil. The plate was then placed on a rocking platform for 10 minutes. The cells were then washed twice in PBS^+ (500 µl/well) and permeabilized using three 5 minute incubations with 500 µl/well of PBS^+ + 0.1% TritonX-100 [Sigma Aldrich, X100] on a rocking platform. After permeabilization, the cells were washed once in ice-cold PBS^+ (500 µl/well) and blocked in 500 µl/well HEK 293 media at room temperature for 30 minutes on a rocking platform. The primary antibody anti-hERG [Santa Cruz, sc-377388] was diluted 1:1000 in ice-cold HEK 293 media and added to the cells (250 µl/well). The plate was then incubated for 1 hour on a rocking platform at room temperature. After incubation, the cells were washed three times in PBS^+ (500 µl/well) before adding the secondary antibody (anti-mouse IgG (H+L) (DyLight 800 conjugate)) [New England Biolabs, 5275S]) diluted 1:1000 in ice-cold HEK 293 media (250 µl/well). After incubation for 1 hour at room temperature on a rocking platform the cells were washed three times in ice-cold PBS^+ (500 µl/well) and the assay signal analysed using a *LI-COR®* Odyssey CLx imaging system.

Analysis and normalization of On/In-Cell Cell Western assay data

To normalise the data generated by the Cell Western assays we used a normalisation by sum approach as outlined by Degasperi *et al.,* [5]. To do this raw arbitrary fluorescence unit intensities (700 and 800 channel values) obtained by the *LI-COR®* Odyssey CLx machine were exported into Excel (Microsoft). The raw *LI-COR®* arbitrary fluorescence unit intensities for the 800 channel were first normalised on a well-by-well basis to the raw *LI-COR®* arbitrary fluorescence unit intensities of the 700 channel that was used to detect the WGA-680 cell stain. The normalized (to cell stain) intensities of the triplicate wells were then averaged. To obtain normalized arbitrary fluorescent unit values the cell-stain normalised averaged values were then normalised to the total summed signal value (800 channel) for that assay.

References

[1] E.K.Emam, S.Rodgers, B.Malin, Anonymising and sharing individual patient data BMJ 350, (2015) h1139.

[2] P.M.Apaja, B.Foo, T.Okiyoneda, W.C.Valinsky, H.Barriere, R.Atanasiu, E.Ficker, G.L.Lukacs, A.Shrier, Ubiquitination-dependent quality control of hERG K+ channel with acquired and inherited conformational defect at the plasma membrane Mol.Biol.Cell 24, (2013) 3787-3804.

[3] Y.Zhang, C.K.Colenso, H.A.El, H.Cheng, H.J.Witchel, C.E.Dempsey, J.C.Hancox, Interactions between amiodarone and the hERG potassium channel pore determined with mutagenesis and in silico docking Biochem.Pharmacol. 113, (2016) 24-35.

[4] D.Melgari, K.E.Brack, C.Zhang, Y.Zhang, H.A.El, J.S.Mitcheson, C.E.Dempsey, G.A.Ng, J.C.Hancox, hERG potassium channel blockade by the HCN channel inhibitor bradycardic agent ivabradine J.Am.Heart Assoc. 4, (2015).

[5] A.Degasperi, M.R.Birtwistle, N.Volinsky, J.Rauch, W.Kolch, B.N.Kholodenko, Evaluating strategies to normalise biological replicates of Western blot data PLoS.One. 9, (2014) e87293.
